# The mechanism underlying the pubertal increase in pulsatile GnRH release in primates

**DOI:** 10.1111/jne.13119

**Published:** 2022-05-01

**Authors:** Ei Terasawa

**Affiliations:** ^1^ Department of Pediatrics and Wisconsin National Primate Research Center University of Wisconsin‐Madison Madison WI USA

**Keywords:** GnRH, human induced pluripotent stem cells, kisspeptin, NKB

## Abstract

In primates, the gonatotropin‐releasing hormone (GnRH) neurosecretory system, consisting of GnRH, kisspeptin, and neurokinin B neurons, is active during the neonatal/early infantile period. During the late infantile period, however, activity of the GnRH neurosecretory system becomes minimal as a result of gonadal steroid independent central inhibition, and this suppressed GnRH neurosecretory state continues throughout the prepubertal period. At the initiation of puberty, the GnRH neurosecretory system becomes active again because of the decrease in central inhibition. During the progress of puberty, kisspeptin and neurokinin B signaling to GnRH neurons further increases, resulting in the release of gonadotropins and subsequent gonadal maturation, and hence puberty. This review further discusses potential substrates of central inhibition and subsequent pubertal modification of the GnRH neurosecretory system by the pubertal increase in steroid hormones, which ensures the regulation of adult reproductive function.

## 1 INTRODUCTION

Unlike the neurohypophysis, the adenohypophysis does not receive direct innervation from the hypothalamus. Yet, the hypothalamus influences the secretory activity of adenohypophyseal hormones luteinsing hormone (LH) and follicle‐stimulating hormone (FSH), which are indispensable for puberty onset and maintenance of reproductive function. As early as early last century, the concept that sexual maturation is controlled by the central nervous system has been proposed, as a result of a clinical observation showing that delayed puberty in patients with Frohlich's syndrome is associated with damage of the ventral region of the brain.^1^ Three decades later, Hohlweg and Junkmann^2^ advanced the concept that a neural sex center is involved in controlling puberty and reproductive cyclicity. Meanwhile, during the 1940s to 1950s, Everett^3^ and Sawyer^4^ showed the influence of the hypothalamus on cyclic ovulation in rats with neuropharmacological tools in several publications. It was not, however, until 1955 that Harris^5^ showed clear evidence implicating the hypothalamus as central for the initiation of puberty. Harris and Jacobson^6^ demonstrated that either prepubertal pituitary glands transplanted into adult hypophysectomized rats or immature ovaries grafted into adult ovariectomized rats were able to sustain estrous cyclicity, whereas grafting adult pituitary gland or ovaries into sexually immature hypophysectomized or ovariectomized rats failed to maintain the estrous cyclicity. Finally, in 1971, two competitive research groups, comprising Amoss et al.^7^ and Matsuo et al.^8^ isolated and sequenced the mammalian form of the gonadotropin‐releasing hormone (GnRH) peptide.

Puberty is one of the most prominent developmental events in life. During puberty, major hormonal, morphological, physiological, and behavioral changes occur such that after puberty the full capacity of reproductive function is established. Because puberty is a transitional period between childhood and adulthood, in primates it takes a couple to several years from beginning to completion. Normal timing of puberty onset is particularly important because precocious puberty not only impairs physical growth, but also leads to higher risks of diseases such as cancers, hypertension, diabetes, hypercholesterolemia, and metabolic syndrome in adulthood, and delayed puberty influences psychological maturation.^9^, ^10^, ^11^, ^12^ During the past 50 years, we have attained direct evidence showing that an increase in pulsatile GnRH release is the key mechanism of puberty onset and have demonstrated how activity of the GnRH neuron increases during the transitional period along with changes in upstream regulatory neuronal systems. This review summarizes what we know about the mechanism of the pubertal increase in GnRH release in primates.

## 2 ONTOGENY OF GNRH NEURONS AND CHANGES IN GNRH RELEASE PATTERN DURING EMBRYONIC DEVELOPMENT

**FIGURE 1:**
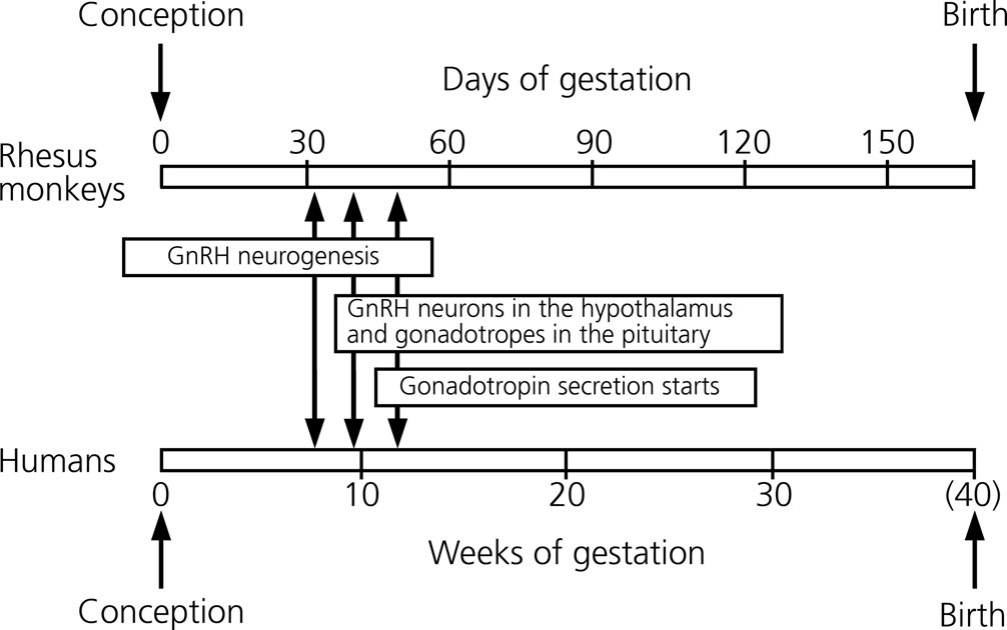
Ontogeny of gonatotropin‐releasing hormone (GnRH) neurons and pituitary‐gonadal axis during the embryonic stage in rhesus monkeys (top) and humans (bottom). Modified from Terasawa.^111^

Unlike most neurons in the brain, mammalian GnRH neurons originate from the nasal epithelium during the early gestational period and migrate into the brain, namely the preoptic area (POA) and medial basal hypothalamus (MBH).^13^, ^14^ In the rhesus macaque, GnRH neurons are found in the nasal placode as early as embryonic day (ED) 32 (E32) and commonly at ED34–36.^15^, ^16^, ^17^, ^18^ GnRH neurons migrate along the nasal septum and then terminal nerve, enter the forebrain through the cribriform sieve at ED38,^16^ and subsequently migrate into the MBH by ED47.^15^ The basic distribution pattern of GnRH neurons in the brain is already established at ED55^16^, ^19^ (Figure 1), although GnRH neurons continue migrating into the POA and MBH until the last trimester. At ED55–70, monkey GnRH neurons are active because gonadotrophs in the pituitary are functional and sex‐specific gonadal steroids are detectable in the umbilical cord at ED70.^20^ Moreover, mRNA expression in GnRH neurons in vitro derived from E36 rhesus macaque embryos dramatically increases after 3 weeks in culture, which is equivalent to approximately ED56–57 in vivo.^21^ The negative feedback loop in the male hypothalamic‐pituitary‐gonadal axis is operative during the second trimester because gonadectomy in male monkeys at ED98–104, but not in female macaques, results in the elevation of LH and FSH.^22^

A similar ontogenic profile of the reproductive neuroendocrine system has been also described in humans.^23^ In human fetuses, GnRH neurons are found in the nasal placode as early as embryonic week (EW) 5.5, although most of them originate in the olfactory pit at EW6.0–6.5. GnRH neurons enter the forebrain through the terminal nerve and then cribiform sieve by EW6.5, and they migrate into the hypothalamus by EW9.0^24^, ^25^ (Figure 1). FSH and LH are detectable in the pituitary by EW10, the pituitary starts to respond to GnRH and release gonadotropins into the general circulation by EW11–12, and their content increases until EW25–29. Circulating gonadotropins reach peak levels at mid‐gestation, and both LH and FSH levels subsequently decline during late gestation.^26^, ^27^, ^28^ Interestingly, a sex difference in gonadotropin levels is seen during mid‐gestation^26^, ^29^, ^30^: (1) Pituitary content and circulating concentrations of LH and FSH in female fetuses are higher than those in male fetuses. (2) Circulating testosterone levels are much higher in male fetuses than circulating estradiol levels in female fetuses. The sex difference in gonadotropin levels and the decrease in gonadotropin levels in fetuses during the late gestational period are attributed to the development of the negative feedback mechanism by gonadal steroid hormones from the fetal gonads, as well as from the placenta.^31^

## 3 CHANGES IN GNRH RELEASE AFTER BIRTH THROUGH PUBERTY

### 3.1 Neonatal/infantile period

The direct measurement of GnRH release during the neonatal and early juvenile/prepubertal period has not been conducted. As such, GnRH neural activity in primates during this period are assessed by changes in circulating gonadotropins. In infantile male monkeys, GnRH neurons are active: (1) Circulating LH and testosterone are elevated during the first 2–3 postnatal months^32^, ^33^ and (2) diurnal variations of testosterone are observed.^34^ Importantly, the diurnal variation in infantile males is quantitatively similar to that seen in sexually mature males.^35^ Plant^34^ shows that the hypothalamic‐pituitary axis in males is already fully mature at the neonatal stage because bilateral orchidectomy at 1 week of age results in an increase in LH and FSH secretion, with a pulse pattern very similar to that in castrated adult males.^36^ By contrast, in female monkeys, circulating LH is only slightly elevated during the first 3 months,^33^, ^37^ a moderate elevation of estrogen levels is observed during late gestation through the neonatal period in females,^33^ and ovariectomy in females at 1 week of age induces an attenuated and abbreviated elevation of LH release with slower pulse frequency compared to those in orchidectomized male infants.^37^, ^38^ As such, in females, the negative feedback mechanism appears to be only partially operative in the late gestational period through the neonatal period. Presently, the precise underlying mechanism of the sex difference in activity of GnRH neurons during the neonatal period remains unclear. Both LH and FSH levels in male monkeys decrease after 3–4 months of age.^37^, ^39^, ^40^

Similar to the rhesus macaque, GnRH neurons in human male neonates are already active. LH levels abruptly increase within the first few minutes after birth, followed by an increase in serum concentrations of testosterone during the first 3–21 h.^41^ High levels of LH in the human male infant decline within 6 months and remain low until the time of puberty. FSH levels in human males are slightly elevated for the first three postnatal months, after which they become low.^42^, ^43^ Circulating levels of testosterone are also elevated for 2–4 months postnatally.^44^ By contrast, in female neonates LH levels are only slightly elevated during the first few months of postnatal life, but FSH levels are high for the first 5 months.^42^, ^45^ After the first 6 months of life, circulating levels of FSH, LH and gonadal steroids become low, and the hypothalamic‐pituitary‐gonadal system enters a quiescent stage until the time of puberty. Again, similar to that seen in macaques, the hypothalamic‐pituitary suppression is attributable to non‐gonadal origin because elevated concentrations of LH and FSH in infants with gonadal dysgenesis declines when they reach the juvenile period, as seen in eugonadal children.^43^, ^46^, ^47^ The transient increase in circulating gonadotropin and gonadal steroids during the neonatal period is also called “mini‐puberty”. Physiological significance of mini‐puberty in males is two fold: (1) transient elevation of testosterone would be important for development of male genitalia, such as penile and testicular growth as well as the proliferation of gonadic cells in humans^48^, ^49^ and (2) neurobehavioral development in later human life.^50^

### 3.2 Juvenile/prepubertal period

**FIGURE 2:**
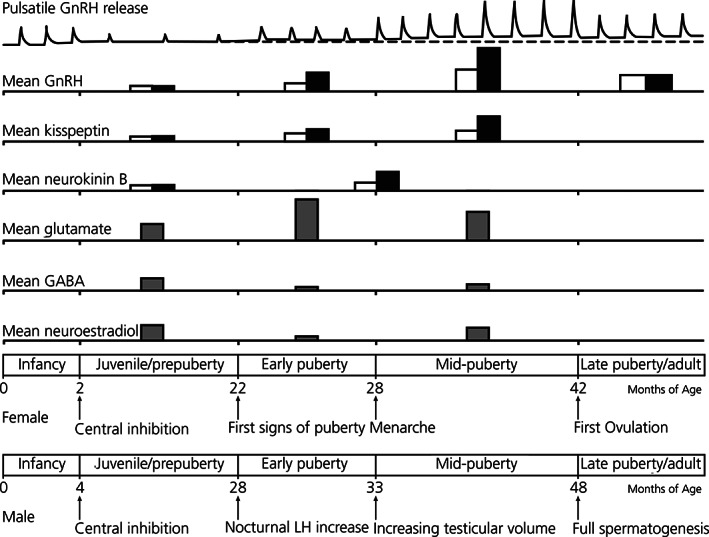
Schematic illustration of postnatal changes in mean release of gonatotropin‐releasing hormone (GnRH), kisspeptin, and neurokinin B (NKB) through puberty in the median eminence of non‐human primates (the second, third and fourth rows^51^, ^52^, ^53^, ^54^). Mean release of glutamate, GABA, and neuroestradiol in the median eminence in females is also shown (fifth, sixth and sevenths rows^80^, ^82^, ^86^, ^115^). All data are extrapolated from the measured values in our previous publications. Note that, although we did not discuss in the text, neuroestradiol is elevated in the macaque median eminence during the prepubertal stage and decreases during the early pubertal stage.^95^ As such, neuroestradiol might be a component of central inhibition in concert with GABA.^115^ The GnRH neurosecretory system is active during the infantile period, but it is suppressed by a central inhibition during the juvenile period, which can be seen as a low frequency and low amplitude of GnRH release (top row^51^, ^52^). Note that mean release of GnRH, kisspeptin, and NKB starts to increase at puberty onset and those changes are further augmented through puberty. A higher evening level of release in GnRH and kisspeptin, shown by filled bars (open bars indicate morning levels) becomes prominent at the time of puberty onset, and the nocturnal increase in GnRH release continues until first ovulation in females, after which GnRH release is reduced to the adult level. In males, the nocturnal increase in GnRH release continues through adulthood. A nocturnal increase in NKB release would occur at puberty onset, but currently we have data from early and midpuberty combined. LH, luteinsing hormone. Modified from Terasawa^116^

Activity of the hypothalamic‐pituitary‐gonadal system is minimal during the prepubertal period. This state of quiescence, unique in primates, represents a period of non‐gonadal inhibition upon GnRH release. Indeed, direct measurements of GnRH release in the hypothalamus indicate that during the entire prepubertal period both the pulse frequency and pulse amplitude of GnRH release are low until the time of puberty in both males and females and regardless of the presence or absence of their gonads (Figure 2).^51^, ^52^, ^53^, ^54^ Importantly, the GnRH neurosecretory system in juvenile monkeys is gonadal steroid independent because developmental reduction in GnRH/LH release is observed in both male and female gonadectomized monkeys^37^, ^40^ and administration of gonadal steroids do not alter either GnRH or gonadotropin levels.^55^ Furthermore, although the GnRH neurosecretory system does not respond to estradiol or androgens, it responds to various neurostimuli, such as electrical stimulation and NMDA challenge. Electrical stimulation of the basal hypothalamus in prepubertal female monkeys results in GnRH release as high as that seen in pubertal female monkeys^56^ and infusion of NMDA into the median eminence (ME) of prepubertal female monkey results in GnRH release.^57^ Importantly, long‐term administration of NMDA at 3 h‐intervals in prepubertal male monkeys results in precocious LH elevation followed by precocious puberty.^58^ Therefore, GnRH neurons are under the central inhibition during the prepubertal period. Presently, the precise mechanism of the central inhibition remains to be investigated. Current knowledge on this topic is discussed in a later section.

In prepubertal humans, both LH and FSH levels are very low. However, results of sensitive assays indicate that during the prepubertal period a minimum GnRH neuronal activity remains because release of LH and FSH is pulsatile and the nocturnal LH and FSH rhythm (i.e., evening levels are higher than those during daytime) is detected.^59^, ^60^ In non‐human primates, although nocturnal increases in GnRH release are seldom seen during the prepubertal period, it is readily observed during the early pubertal period.

### 3.3 Pubertal period

The first hormonal sign of puberty precedes the first physical sign of puberty. At an early phase of puberty (i.e., early pubertal stage), the basal level, pulse frequency and pulse amplitude of LH/FSH release all increase before nipple growth and menarche in girls.^61^ In rhesus females as well, the basal level, frequency, and amplitude of GnRH, LH, and FSH start to increase several months before menarche.^51^ Furthermore, it has been well documented that at an early stage of puberty, nocturnal increase in the LH pulse amplitude becomes prominent in both males and females in humans and rhesus monkeys.^59^, ^62^, ^63^ The pubertal increase in gonadotropin release leads to secretion of estradiol from the ovary and testosterone from the testis, resulting in secondary sex characteristics, such as nipple growth, sex‐skin development, and subsequently, menarche in females. In males, however, external signs of puberty (i.e., an increase in testicular size) are difficult to detect until the mid‐pubertal stage.^54^

During the mid‐pubertal stage, increases in GnRH, LH and estradiol/testosterone accelerate. Although GnRH pulse frequency remains stable after the onset of puberty, the basal level and pulse amplitude of GnRH and LH continue to increase throughout puberty, until first ovulation in females and full spermatogenesis in males occur. During the mid‐pubertal period the nocturnal increases in GnRH release become increasingly prominent.^51^ Importantly, a pubertal increase in GnRH release is independent from the presence or absence of gonadal steroids in circulation in both sexes because release of GnRH and LH in neonatally or prepubertally gonadectomized male and female monkeys also starts to increase at a similar age as gonadally intact counter parts.^40^, ^52^, ^62^

Collectively, active GnRH neurons at the neonatal period are suppressed by central inhibition throughout the prepubertal period until the time of puberty, when a sustained increase in GnRH release, followed by elevated gonadotropin release occurs. The importance of the increased GnRH release at puberty onset is also experimentally shown.^64^ Then, what is the mechanism to initiate puberty?

## 4 NEUROENDOCRINE SIGNALING INITIATING THE PUBERTAL INCREASE IN GNRH RELEASE

**FIGURE 3:**
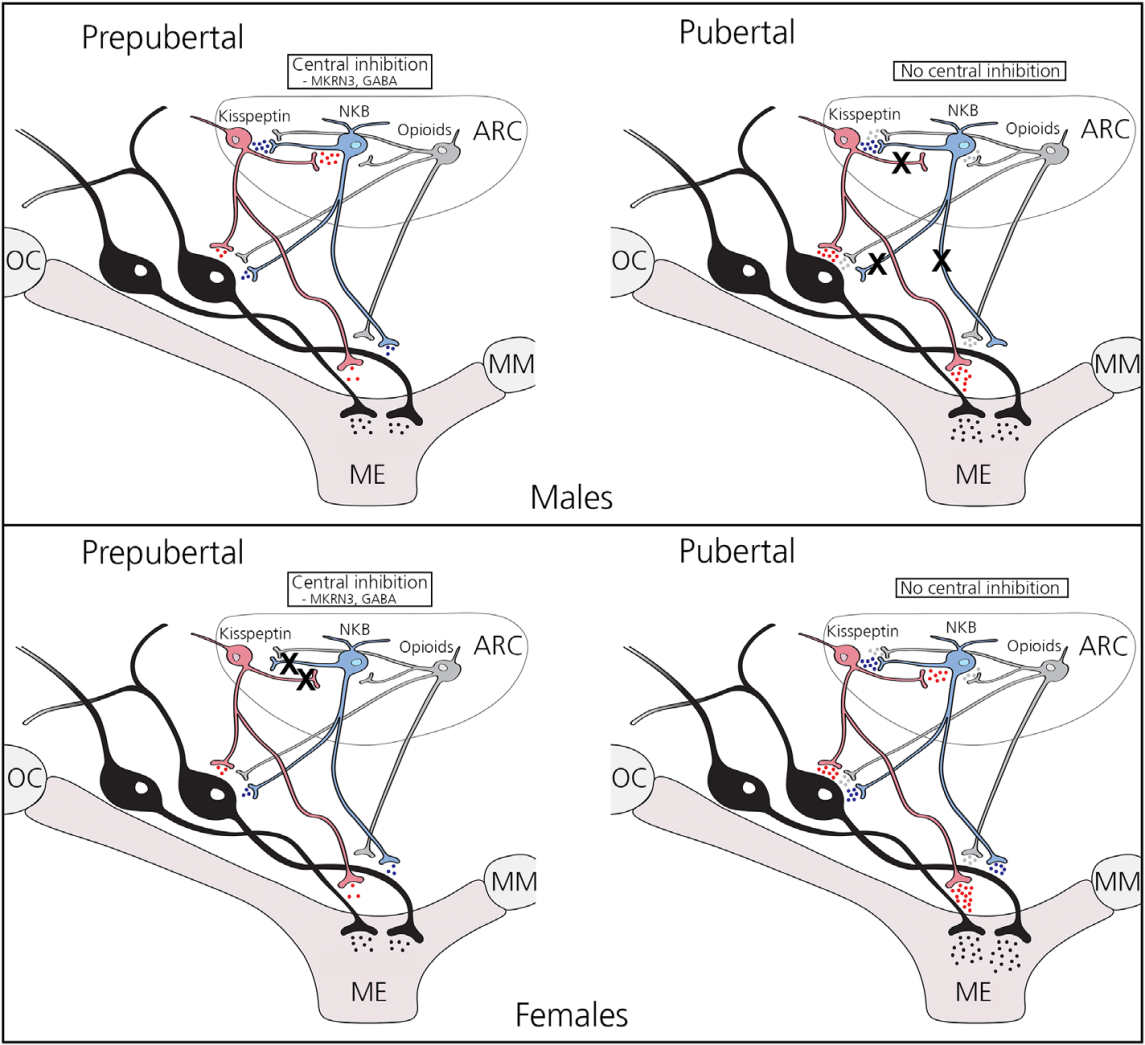
Schematic illustration summarizing developmental changes in the neuroendocrine pathways involved in the mechanism of puberty onset. Possible interactions between kisspeptin (red), neurokinin B (NKB) (blue), opioid (gray), and gonatotropin‐releasing hormone (GnRH) (black) neurons in the hypothalamus in prepubertal and pubertal male (A) and female (B) monkeys are shown. Although, in this schema kisspeptin, NKB, opioid neurons are all clustered in the arcuate nucleus (ARC), interaction between them takes place at the GnRH cell body levels as well as GnRH neuroterminals in the median eminence (ME). The number of colored dots at the neuroterminals reflects the estimated amount of neuropeptide release, based on the results from a series of experiments in this lab.^53^, ^54^, ^84^, ^85^, ^101^, ^102^, ^104^, ^105^, ^116^ A black X indicates signaling pathways are not operative. Note that major remodeling of kisspeptin and NKB signaling pathways takes place during puberty, such that their regulation of the GnRH neurosecretory system is most effective in adult reproductive function. Specifically, in prepubertal males, there is a reciprocal interaction between kisspeptin and NKB signaling (kisspeptin neurons mediate NKB signaling and NKB neurons mediate kisspeptin signaling) and both kisspeptin and NKB signaling modify the activity of GnRH neurons directly and independently. Perhaps the high activity of GnRH neurons during the neonatal period in males reflects the presence of reciprocal pathways in prepubertal males, although subsequent central inhibition during the prepubertal/juvenile keeps GnRH release low (top left). When males reach puberty, direct NKB signaling to GnRH neurons is lost, kisspeptin signaling through NKB neurons is no longer available, and NKB signaling to GnRH neurons needs to be mediated through kisspeptin neurons (top right). It is speculated that a simple NKB upstream signaling mechanism mediated through kisspeptin neurons is sufficient in the regulation of GnRH release in adult male reproductive function. By contrast, in prepubertal females, although both kisspeptin and NKB signaling can directly and independently influence activity of GnRH neurons, there is no reciprocal interaction between kisspeptin and NKB signaling (bottom left). In pubertal females, however, kisspeptin and NKB signaling not only influence GnRH neurons directly and independently, but also new reciprocal interactions between kisspeptin and NKB signaling are established (bottom right). These reciprocal and cooperative kisspeptin and NKB signaling pathways would provide more power and flexibility to regulate GnRH neurons in adult females, such that cyclic ovulations and pregnancy can be achieved. The role of opioid neurons is rather hypothetical at this point. Adapted^54^, ^103^, ^109^ and reproduced from Terasawa.^102^ GABA, gamma aminobutyric acid; ME, median eminence; MKRN3, the gene encoding makorin RING‐finger protein 3; MM, mammillary body; OC, optic chiasm

Clinical studies in human genetics indicate that kisspeptin and neurokinin B (NKB) signaling play critical roles in puberty, as patients with mutations in the genes encoding *KISS1* or *NK3* and their receptors, KISS1R or NK3R, respectively, exhibit abnormal timing of puberty or no puberty.^65^, ^66^, ^67^ Subsequent studies in animal experiments suggest that (1) kisspeptin neurons express estrogen receptor alpha (ERα); (2) kisspeptin neurons in the anterior ventral nucleus (AVPV) innervate cell bodies of GnRH neurons, whereas kisspeptin neurons in the arcuate nucleus (ARC) innervate GnRH neuroprocesses; and (3) kisspeptin is an upstream regulator of basal as well as preovulatory GnRH release.^68^, ^69^, ^70^, ^71^ Herde et al.^72^ renamed GnRH neuroprocesses in the ME as “dendrones” because these neuroprocesses have properties of both dendrites and axons. Moreover, because a subset of kisspeptin neurons in the ARC of the mouse and sheep co‐expresses NKB and dynorphin,^73^ Goodman and colleagues have named them “KNDy neurons”.^74^, ^75^, ^76^, ^77^ In primates, however, only a small subset of kisspeptin neurons in the ARC co‐express both NKB and dynorphin^78^, ^79^ and co‐expression of kisspeptin, NKB, and dynorphin within a single ARC neuron may not be critical for pulse‐generation because interaction between the three peptides could occur at their neuroterminals. As such, in this review, we discuss these three types of neurons, as the independent units forming the regulatory network for GnRH release (Figure 3).

Our recent studies suggest that during the prepubertal period the release of NKB and kisspeptin neurons is also very low and release of both peptides increases along with the pubertal increase in GnRH release (Figure 2, ^53^; also unpublished observations by JP Garcia and E Terasawa). Therefore, central inhibition governs the entire GnRH neurosecretory system and the removal of central inhibition is essential for the onset of puberty.

### 4.1 Central inhibition

Underlying mechanisms and neuronal substrates composing central inhibition are not well understood. Many years ago, we proposed the hypothesis that the GABA neuron is a part of the neural substrate of central inhibition. This hypothesis is based on the observations that (1) GABA release in the ME in prepubertal monkeys was higher than that in early‐ and mid‐pubertal monkeys^80^; (2) infusion of the GABA_A_ receptor blocker, bicuculline, into the ME stimulates GnRH release in prepubertal, but not pubertal monkeys^80^; (3) infusion of antisense oligonucleotides to the rate limiting enzyme for GABA synthesis, GAD‐67 and GAD‐65 mRNAs, into the ME of prepubertal monkeys resulted in a large increase in GnRH release, whereas the same procedure in pubertal monkeys stimulated only a small increase in GnRH release^81^, ^82^; and finally (4) pulsatile infusion of bicuculline into the base of the third ventricle results in precocious menarche and first ovulation.^83^ More recent studies further indicate that infusion of bicuculline into the ME stimulates kisspeptin release^84^ and NKB release^85^ in prepubertal, but not pubertal monkeys. Importantly, the results from a follow‐up study of the antisense mRNA infusion study that a decrease in GABA synthesis by interference with GAD67 synthesis followed by the reduction in GABA release triggers an increase in GnRH release accompanied with increase in glutamate release, suggesting the contribution of glutamate for the pubertal increase in GnRH release.^86^ A similar prepubertal GABA dominant inhibitory mechanism appears to exist in male macaques because a recent study indicates that the tonic GABA inhibition over release of kisspeptin and NKB in the ME was observed in prepubertal, not pubertal, male rhesus monkeys.^85^ Collectively, it appears that GABA and glutamate neurons located up‐stream of the kisspeptin and NKB neurons are a part of neuronal circuits involved in central inhibition.

Because the direct action of GABA is stimulatory for the GnRH neuron,^87^ clarification may be needed regarding the consistent findings on the inhibitory role of GABA in pubertal GnRH neurons. Although the stimulatory action of GABA on GnRH neurons is shown by recording from single cell studies with anatomically identified GnRH neurons,^87^ in our studies,^80^, ^81^, ^82^, ^83^, ^84^, ^85^ inhibitory action of GABA is derived from the direct measurement of GnRH release in the hypothalamus under the presence of GABA or GABA receptor agonists and antagonists in prepubertal macaques. As such, GABA action on GnRH release that we described is involved in multiple neural pathways. Importantly, the inhibitory role of GABA is seen in prepubertal but not pubertal monkeys,^80^, ^81^, ^82^ whereas excitatory action of GABA on mouse GnRH neurons occurs throughout the developmental stages,^88^ clearly suggesting that the neural pathway can be changed across puberty, but direct action of GABA is consistently stimulatory. Therefore, the difference between “inhibitory” versus “excitatory” role of GABA in GnRH neurons in the context stems from differences in experimental approaches.

In 2013, a striking report indicative of the mechanism of central inhibition was published. In their paper, Abreu et al.^89^ reported that human patients with frame shift mutations of *MKRN3*, the gene encoding makorin RING‐finger protein 3, exhibit central precocious puberty and that MKRN3 mRNA expression in the ARC of male and female mice was higher on postnatal day (P)10 (P10) than P20. Importantly, the mutation in *MKRN3* occurs at the zinc‐finger domain, consistent with reports of gene‐wide genome association studies regarding gene associated with the age of puberty.^89^, ^90^, ^91^ Involvement of zinc‐finger protein‐mediated transcriptional repression of GnRH neurons as the central inhibition has also been reported in prepubertal monkeys.^92^ A more recent study by Abreu et al.^93^ further indicates that MKRN3 mRNA expression in the female rhesus monkey hypothalamus at less than 6 months of age were highest and gradually decreased at 6–12 months reaching the lowest at 12–30 months of age, and MKRN3 mRNA expression in the hypothalamus of both male and female mice was gonadal steroid independent because the *hpg* mouse that lacks active gonads exhibits a similar developmental decrease in MKRN3 mRNA expressions in the whole hypothalamus. It was further shown that the developmental decrease in MKRN3 mRNA also occurs in the ARC and AVPV of mice in both sexes and that MKRN3 mRNA is colocalized in mice kisspeptin neurons in the ARC and AVPV. Finally, the it was shown that MKRN3 inhibited Kiss1 and Tac3 promoter activity by repressing KISS1 and TAC3 transcription. Together, it was proposed the hypothesis that MKRN3 represses activity of kisspeptin and NKB neurons through an MKRN3‐directed ubiquitination‐mediated mechanism and developmental decrease in MKRN3 activity results in disinhibition of GnRH neurons, resulting in the pubertal increase in the GnRH peptide.^93^

The study by Abreu et al.^93^ is one of the most impressive works in recent years. However, because the assessment of changes in MKRN3 mRNA in the monkey study was conducted with ill‐defined developmental stages in females and Abreu et al.^93^ did not confirm changes in MKRN3 mRNA with developmental changes in hormones (LH, FSH, and gonadal steroids), it is difficult to apply the concept to monkey puberty at this time. For example, grouping monkeys at 0–6 months of age when the highest MKRN3 mRNA level seen means that this group includes the brains from monkeys at “neonatal mini‐puberty” and the beginning of the prepubertal period. Similarly, grouping female monkeys at 12–30 months of age means females at prepubertal and early pubertal period are grouped together. An additional study with more precise age groups in both sexes independently with monitoring gonadotropin levels is clearly needed. This fundamental flaw aside, however, many questions arise. First, it is unclear whether the mechanism observed in mice is applicable to primate puberty, including humans: (1) the time course between birth and puberty onset in primates is much longer than in mice and (2) convincing evidence for central inhibition in the mouse has not been reported, and in the mouse, gonadal steroid sensitive GnRH suppression dominates prior to puberty onset^94^; there is also a species difference issue.^95^ Second, as discussed earlier, at the neonatal period GnRH release and gonadotropin secretion in primates are elevated, resulting in “mini‐puberty”. If MKRN3 mRNA levels are maximally elevated in animals at the neonatal period, how can high activity of GnRH neurons be achieved? Third, assuming a similar mechanism to that seen in mice can take place in primates, it is still unclear what triggers a decrease in MKRN3 mRNA in neurons of the ARC and AVPV? Fourth, does decrease in MKRN3 mRNA in the ARC and AVPV play a similar role in both sexes? Unlike that seen in rodents, there is little sex difference in the function of AVPV kisspeptin neurons in primates,^96^, ^97^ although it is known that male puberty occurs much later than in female puberty in both species. Fifth, as discussed above, a series of studies^80^, ^81^, ^82^, ^83^, ^84^, ^85^ consistently indicate that the GABA neuron is a part of neuronal circuits for central inhibition in non‐human primates. Then, how do changes in GABA neurons at the onset of puberty relate to the developmental changes in the ubiquitin ligase MKRN3 in kisspeptin and NKB neurons? Presently, we have more questions than answers.

Note that there are consistent reports showing that neuropeptide Y (NPY) also plays a role in central inhibition in male monkeys.^98^, ^99^ Because GABA and NPY are colocalized in ARC neurons,^100^ there is a possible common mechanism between GABA and NPY in central inhibition. This hypothesis needs to be tested in a series of future studies.

### 4.2 Activity of kisspeptin and NKB neurons increases at puberty onset

After the reduction of central inhibition, activity of kisspeptin and NKB signaling over GnRH neurons increases. We have systematically examined the manner in which kisspeptin and NKB signaling stimulates the pubertal increase in GnRH release in non‐human primates. As described below, kisspeptin and NKB signaling accelerates the pubertal increase in GnRH release through two mechanisms: (1) kisspeptin and NKB neurons release a higher amount of respective peptides and (2) an increased sensitivity of KISS1R on GnRH neurons.

#### 4.2.1 Kisspeptin release

Kisspeptin release in the ME is pulsatile and the pulse frequency is similar to that in GnRH release. Indeed, kisspeptin pulses are highly correlated to GnRH pulses.^101^ There is a clear developmental change in kisspeptin release: Although, in prepubertal female monkeys, the mean release, pulse frequency, and pulse amplitude of kisspeptin release are all low, in pubertal females, all components of kisspeptin release significantly increase^53^ (Figure 2). Importantly, in an early phase after puberty onset, the pubertal increase in kisspeptin release is gonadal steroid independent because an increase in kisspeptin release also occurs in gonadectomized animals,^53^ but the later phase of the kisspeptin increase is partly a result of the pubertal increase in circulating gonadal steroid hormones.^53^, ^102^ In males, kisspeptin release also gradually increases after puberty onset.^103^

#### 4.2.2 NKB release

NKB release in the ME of prepubertal males is low and it increases at puberty onset. Again, the pubertal increase in NKB release in males is gonadal steroid independent (JP Garcia and E Terasawa, unpublished observations). Whether the pubertal increase in NKB release occurs in females has not been examined. As seen in kisspeptin release, we assume that NKB release is pulsatile, although we have not confirmed this systematically yet.

### 4.3 Changes in the sensitivity of GnRH neurons to kisspeptin and NKB signaling

In female monkeys GnRH neurons respond to both kisspeptin (kisspeptin10) and NKB (senktide) signaling in a dose‐responsive manner.^53^, ^104^ Because the GnRH response to the same dose of kisspeptin10 in pubertal females is larger than in prepubertal females, the sensitivity of GnRH neurons to kisspeptin signaling clearly increases after puberty onset. Importantly, the pubertal increase in responsiveness of GnRH neurons to kisspeptin signaling is a result of the pubertal increase in circulating gonadal steroids.^104^ By contrast, in males, although both kisspeptin and NKB are stimulatory to GnRH release and the response of GnRH neurons to kisspeptin (kisspeptin10) signaling is dose‐dependent, the GnRH response to NKB signaling is not dose‐dependent.^54^, ^102^, ^103^ Moreover, in male monkeys, developmental amplification of the GnRH response to kisspeptin10 is seen only at a higher dose.^103^ Interestingly, GnRH neurons in females are more sensitive to kisspeptin signaling than in males because a 1/100‐fold smaller dose of kisspeptin induces a larger response in females than in males at both the prepubertal and pubertal stages.^102^, ^103^ The underlying mechanism of the sex difference in GnRH response to kisspeptin signaling is currently unknown.

### 4.4 Kisspeptin and NKB signaling pathways to GnRH neurons undergo pubertal change

Although it is well documented that GnRH neurons express kisspeptin receptors^105^, ^106^ and kisspeptin directly modulates GnRH release, the direct action of NKB on GnRH release remains controversial. NKB neurons, however, can modulate GnRH neurons directly through dendrones because GnRH neuroterminal fibers in the ME express NK3R,^107^ and a study using fast scan cyclic voltammetry with a carbon‐fiber microelectrode indicates that the direct action of the NK3R agonist senktide on GnRH neurons occurs in the neuroterminal region, but not in the POA.^108^ Therefore, microdialysis experiments examining interactions between kisspeptin and NKB signaling to GnRH release conducted in the ME in our laboratory are physiologically relevant.

To clarify the hierarchy between kisspeptin and NKB signaling that influences activity of GnRH neurons, we have conducted a series of experiments using agonists and antagonists for KISS1R and NK3R. Through such experiments, we have observed quite surprising results: Kisspeptin and NKB signaling pathways to GnRH neurons undergo pubertal change in both males and females, and the direction of the pathway changes in the two sexes is almost opposite. Because detailed experimental designs and results have been published previously,^103^, ^104^, ^109^ in this review, we just summarize the results and interpretations. In prepubertal males, there is a reciprocal interaction between kisspeptin and NKB signaling (kisspeptin neurons mediate NKB signaling and NKB neurons mediate kisspeptin signaling) and both kisspeptin and NKB signaling modify the activity of GnRH neurons directly and independently. We speculate that the powerful reciprocal pathways in prepubertal males might reflect the high activity of GnRH neurons during the neonatal period in males but subsequent Central Inhibition during the prepubertal/juvenile keeps GnRH release low (Figure 3, top left). When males reach puberty (i.e., in pubertal males), direct NKB signaling to GnRH neurons is lost, kisspeptin signaling through NKB neurons is no longer available, and NKB signaling to GnRH neurons needs to be mediated through kisspeptin neurons (Figure 3, top right). The upstream regulation of NKB signaling through kisspeptin neurons has also been reported in male monkeys.^110^ Perhaps a simple mechanism underlying NKB signaling mediated through kisspeptin neurons is sufficient in the regulation of GnRH release, and hence male reproductive function in adulthood. By contrast, in prepubertal females, although both kisspeptin and NKB signaling can directly and independently influence activity of GnRH neurons, there is no reciprocal interaction between kisspeptin and NKB signaling (Figure 3, bottom left). In pubertal females, however, kisspeptin and NKB signaling not only influences GnRH neurons directly and independently, but also new reciprocal interactions between kisspeptin and NKB signaling are established (Figure 3, bottom right). These reciprocal and cooperative kisspeptin and NKB signaling pathways would provide more power and flexibility to regulate GnRH neurons in adult females, such that cyclic ovulations and pregnancy can be achieved.

In summary, we now know that during the postnatal development, a sustained elevation of pulsatile GnRH release results in puberty in both males and female primates. Indeed, transient elevation of the GnRH release does not lead to puberty. As described earlier, the GnRH neurosecretory system in males shortly after birth is fully active, similar to that observed in adulthood, but, at the late neonatal period, central inhibition comes in, suppressing the elevated GnRH neurosecretory activity. In females as well, a similar, but smaller degree of the transient GnRH elevation is seen during the early neonatal period, and central inhibition comes in, suppressing the GnRH neurosecretory system. Although the neural substrates responsible for central inhibition remain unclear, the suppression of GnRH release continues throughout the prepubertal period. It is considered that central inhibition for reproductive function provides the time for maturation of higher brain function, including intellectual, judgement, memory function, and sensory‐motor function.

Recent studies^54^, ^85^, ^103^ investigating the role of the KNDy neurons forming the GnRH neurosecretory system further suggest that the reduction in central inhibition comes first. During the early stage of the pubertal period, active interaction is observed between kisspeptin and NKB signaling to GnRH neurons, which is reminiscent of the very active GnRH neurosecretory system during the neonatal period in males, whereas, during the late pubertal period, a simple NKB dominant pathway to GnRH neurons (i.e., NKB signaling → kisspeptin signaling → GnRH neurons) is established, such that male adult reproductive function does not require complex neuroendocrine mechanisms. By contrast, whereas, during the early stage of pubertal period in females, there is no cooperative interaction between kisspeptin and NKB signaling to GnRH neurons, during the late pubertal period to adulthood, a flexible and cooperative relationship between kisspeptin and NKB signaling is established to be suitable for adult female reproductive function. Therefore, the pubertal period can be redefined as the time when the GnRH neurosecretory system is reorganized/remodeled to tailor the need for adult reproductive function.

Finally, brief comments on the role of opioid neurons including dynorphin neurons in the pubertal increase in pulsatile GnRH. We already know that opioid neurons are not involved in central inhibition,^111^ whereas the preliminary data indicate that release of β‐endorphin increases after puberty onset.^112^ Therefore, we speculate that opioid peptides play an important role for the pubertal increase in pulsatile GnRH release, as a part of the break. Indeed, a recent report showed that tonic infusion of the opioid antagonist naloxone increased the LH pulse frequency in patients with deficiency of NKB signaling genes,^113^ indicating the role of opioid neurons in GnRH pulse generation. Future studies warrant clarification of the role of opioid neurons in the pubertal increase in GnRH release.

## 5 CONCLUSIONS AND FUTURE PERSPECTIVES

Since the discovery of the GnRH molecule in 1971,^7^, ^8^ great advancements have been made in neuroendocrine research. Specifically, the isolation and molecular identification of the GnRH molecule from the hypothalamus, followed by the discovery of many other small peptides including kisspeptin, NKB, and several opioids in the brain, have played a great role. Although, in this article the author did not discuss non‐peptidergic neurotransmitters, such as acetylcholine, serotonin, dopamine, epinephrine, norepinephrine, and nitric oxide, they are also known to modulate GnRH neuronal activity directly or indirectly. Nevertheless, the concept that an increase in pulsatile GnRH release initiates puberty, and the pubertal increase in GnRH release is accompanied by the increased activity of kisspeptin and NKB neurons, is firmly established. We now know that the presence and proper connections between GnRH, kisspeptin and NKB neurons in the hypothalamus are all necessary for puberty onset and subsequent maintenance of normal reproduction functions. Neural substrates and mechanisms of central inhibition remain to be investigated, but the availability of various in vivo and in vitro models including GnRH neurons derived from human stem cells^114^ would provide a bright future for research in GnRH neurobiology.

## CONFLICTS OF INTEREST

The author declares that she has no conflicts of interest.

## AUTHOR CONTRIBUTIONS

The manuscript has been designed and written by Ei Terasawa.

### PEER REVIEW

The peer review history for this article is available at https://publons.com/publon/10.1111/jne.13119.

## Data Availability

Data sharing is not applicable to this article as no new data were created or analyzed in this study.

## References

[1] Erdheim J . Über hypophysenganggeschwulste und hirmcholesteatome. Sitzungsb Kais Akad Wissen Math Naturw Klin. 1904;113:537‐726.

[2] Hohlweg W , Junkmann K . Die hormonal‐nervose regulierung der funktion des hypophysenvorderlappens. Klin Wochenschr. 1932;11:321‐323.

[3] Everett JW . Neurobiology of reproduction in the female rat. A fifty‐year perspective. Monogr Endocrinol. 1989;32:1‐133. 2695836

[4] Sawyer CH . First Geoffrey Harris Memorial lecture. Some recent developments in brain‐pituitary‐ovarian physiology. Neuroendocrinology. 1975;17:97‐124. 1094317 10.1159/000122347

[5] Harris GW . Neural Control of the Pituitary Gland. London, UK: Edward Arnold; 1955.

[6] Harris GW , Jacobson D . Functional grafts of the anterior pituitary gland. Proc R Soc London B: Biol Sci. 1952;139:263‐276. 14911829 10.1098/rspb.1952.0011

[7] Amoss M , Burgess R , Blackwell P , Vale W , Fellows R , Guillemin R . Purification amino acid composition and N‐terminus of the hypothalamic luteinizing hormone releasing factor (LRF) of ovine origin. Biochem Biophys Res Commun. 1971;44:205‐210. 4940370 10.1016/s0006-291x(71)80179-1

[8] Matsuo H , Baba Y , Nair RMG , Arimura A , Schally AV . Structure of the porcine LH‐ and FSH‐releasing hormone. I. The proposed amino acid sequence. Biochem Biophys Res Commun. 1971;43:1334‐1339. 4936338 10.1016/s0006-291x(71)80019-0

[9] Hamilton AS , Mack TM . Puberty and genetic susceptibility to breast cancer in a case‐control study in twins. N Engl J Med. 2003;348:2313‐2322. 12788995 10.1056/NEJMoa021293

[10] Wardle J , Brodersen NH , Cole TJ , Jarvis MJ , Boniface DR . Development of adiposity in adolescence: five year longitudinal study of an ethnically and socioeconomically diverse sample of young people in Britain. BMJ. 2006;332:1130‐1135. 16679329 10.1136/bmj.38807.594792.AE PMC1459611

[11] Jasik CB , Lustig RH . Adolescent obesity and puberty: the "perfect storm". Ann N Y Acad Sci. 2008;1135:265‐279. 18574233 10.1196/annals.1429.009

[12] Dwyer AA . Psychosexual effects resulting from delayed, incomplete, or absent puberty. Curr Opin Endocr Metab Res. 2020;14:15‐21. 32524038 10.1016/j.coemr.2020.04.003 PMC7286539

[13] Schwanzel‐Fukuda M , Pfaff DW . Origin of luteinizing hormone‐releasing hormone neurons. Nature. 1989;338:161‐164. 2645530 10.1038/338161a0

[14] Wray S , Grant P , Gainer H . Evidence that cells expressing luteinizing hormone‐releasing hormone mRNA in the mouse are derived from progenitor cells in the olfactory placode. Proc Natl Acad Sci U S A. 1989;86:8132‐8136. 2682637 10.1073/pnas.86.20.8132 PMC298229

[15] Ronnekleiv OK , Resko JA . Ontogeny of gonadotropin releasing hormone‐containing neurons in early fetal development of rhesus macaques. Endocrinology. 1990;126:498‐511. 2104589 10.1210/endo-126-1-498

[16] Quanbeck C , Sherwood NM , Millar RP , Terasawa E . Two populations of luteinizing hormone‐releasing hormone neurons in the forebrain of the rhesus macaque during embryonic development. J Comp Neurol. 1997;380:293‐309. 9087514

[17] Terasawa E , Quanbeck CD , Schulz CA , Burich AJ , Luchansky LL , Claude P . A primary cell culture system of luteinizing hormone releasing hormone neurons derived from embryonic olfactory placode in the rhesus monkey. Endocrinology. 1993;133:2379‐2390. 8404690 10.1210/endo.133.5.8404690

[18] Terasawa E , Keen KL , Mogi K , Claude P . Pulsatile release of luteinizing hormone‐releasing hormone (LHRH) in cultured LHRH neurons derived from the embryonic olfactory placode of the rhesus monkey. Endocrinology. 1999;140:1432‐1441. 10067872 10.1210/endo.140.3.6559

[19] Terasawa E , Busser BW , Luchansky LL , et al. Presence of luteinizing hormone‐releasing hormone fragments in the rhesus monkey forebrain. J Comp Neurol. 2001;439:491‐504. 11596068 10.1002/cne.1364

[20] Resko JA , Ellinwood WE , Pasztor LM , Buhl AE . Sex steroids in the umbilical circulation of fetal rhesus monkeys from the time of gonadal differentiation. J Clin Endocrinol Metab. 1980;50:900‐905. 6768761 10.1210/jcem-50-5-900

[21] Kurian JR , Keen KL , Terasawa E . Epigenetic changes coincide with in vitro primate GnRH neuronal maturation. Endocrinology. 2010;151:5359‐5368. 20861233 10.1210/en.2010-0555 PMC2954729

[22] Resko JA , Ellinwood EE . Negative feedback regulation of gonadotropin secretion by androgens in fetal rhesus macaques. Biol Reprod. 1985;3:346‐352. 10.1095/biolreprod33.2.346 3929848

[23] Grumbach MM , Kaplan SL . The neuroendocrinology of human puberty, an ontogenetic perspective. In: Grumbach MM , Sizonenko PC , Aubert ML , eds. Control of the Onset of Puberty. Philadelphia, PA: Williams & Wilkins; 1990:1‐62.

[24] Schwanzel‐Fukuda M , Crossin KL , Pfaff DW , Bouloux PM , Hardelin JP , Petit C . Migration of luteinizing hormone‐releasing hormone (LHRH) neurons in early human embryos. J Comp Neurol. 1996;366:547‐557. 8907364 10.1002/(SICI)1096-9861(19960311)366:3<547::AID-CNE12>3.0.CO;2-M

[25] Verney C , el Amraoui A , Zecevic N . Comigration of tyrosine hydroxylase‐ and gonadotropin‐releasing hormone‐immunoreactive neurons in the nasal area of human embryos. Brain Res Dev Brain Res. 1996;97:251‐259. 8997509 10.1016/s0165-3806(96)00147-2

[26] Grumbach MM , Roth JC , Kaplan SL , Kelch RP . Hypothalamic‐pituitary regulation of puberty in man: evidence and concepts derived from clinical research. In: Grumbach MM , Grave GD , Mayer FE , eds. Control of the Onset of Puberty. New York, NY: John Wiley & Sons; 1974:115‐166.

[27] Kaplan SL , Grumbach MM , Aubert ML . The ontogenesis of pituitary hormones and hypothalamic factors in the human fetus: maturation of central nervous system regulation of anterior pituitary function. Recent Prog Horm Res. 1976;32:161‐243. 785555 10.1016/b978-0-12-571132-6.50015-4

[28] Clements JA , Reyes FI , Winter JS , Faiman C . Studies on human sexual development. III. Fetal pituitary and serum, and amniotic fluid concentrations of LH, CG, and FSH. J Clin Endocrinol Metab. 1976;42:9‐19. 1249196 10.1210/jcem-42-1-9

[29] Winter JSD , Faiman C , Reyes FI . Sex steroid production by the human fetus: its role in morphogenesis and control by gonadotropins. Birth Defects Orig Artic Ser. 1977;13:41‐58. 213140

[30] Castillo RH , Matteri RL , Dumesic DA . Luteinizing hormone synthesis in cultured fetal human pituitary cells exposed to gonadotropin‐releasing hormone. J Clin Endocrinol Metab. 1992;75:318‐322. 1619025 10.1210/jcem.75.1.1619025

[31] Styne DM , Grumbach MM . Puberty, ontogeny, nuroendocrinology, physiology, and disorders. In: Kroenenberg H , Melmed S , Polonsky KS , Larsen PR , eds. Williams Textbook of Endocrinology. 11th ed. Philadelphia, PA: Saunders Elsevier; 2008:970‐1166.

[32] Frawley LS , Neill JD . Age related changes in serum levels of gonadotropins and testosterone in infantile male rhesus monkeys. Biol Reprod. 1979;20:1147‐1151. 113042 10.1095/biolreprod20.5.1147

[33] Robinson JA , Bridson WE . Neonatal hormone patterns in the macaque. I. Steroids. Biol Reprod. 1978;19:773‐778. 105767 10.1095/biolreprod19.4.773

[34] Plant TM . Neuroendocrine basis of puberty in the rhesus monkey (*Macaca mulatta*). In: Martini L , Ganong WF , eds. Frontiers in Neuroendocrinology. Raven Press; 1988:215‐238.

[35] Plant TM . Pulsatile luteinizing hormone secretion in the neonatal male rhesus monkey (*Macaca mulatta*). J Endocrinol. 1982;93:71‐74. 7069347 10.1677/joe.0.0930071

[36] Plant TM . A striking diurnal variation in plasma testosterone concentrations in infantile male rhesus monkeys (*Macaca mulatta*). Neuroendocrinology. 1982;35:370‐373. 7145027 10.1159/000123409

[37] Pohl CR , de Ridder CM , Plant TM . Gonadal and nongonadal mechanisms contribute to the prepubertal hiatus in gonadotropin secretion in the female rhesus monkey (*Macaca mulatta*). J Clin Endocrinol Metab. 1995;80:2094‐2101. 7608261 10.1210/jcem.80.7.7608261

[38] Plant TM . A striking sex difference in the gonadotropin response to gonadectomy during infantile development in the rhesus monkey (*Macaca mulatta*). Endocrinology. 1986;119:539‐545. 3089758 10.1210/endo-119-2-539

[39] Steiner RA , Bremner WJ . Endocrine correlates of sexual development in the male monkey, *Macaca fascicularis* . Endocrinology. 1981;109:914‐919. 7021130 10.1210/endo-109-3-914

[40] Plant TM . A study of the role of the postnatal testes in determining the ontogeny of gonadotropin secretion in the male rhesus monkey (*Macaca mulatta*). Endocrinology. 1985;116:1341‐1350. 3971918 10.1210/endo-116-4-1341

[41] Corbier P , Dehennin M , Castanier A , Mebazaa A , Edwards DA , Roffi J . Sex differences in serum luteinizing hormone and testosterone in the human neonate during the first few hours after birth. J Clin Endocrinol Metab. 1990;71:1344‐1348. 2229291 10.1210/jcem-71-5-1344

[42] Faiman C , Winter JSD . Gonadotropins and sex hormone patterns in puberty, clinical data. In: Grumbach MM , Grave GD , Mayer FE , eds. Control of the Onset of Puberty. New York, NY: Wiley; 1974:32‐55.

[43] Winter JSD , Faiman C . Serum gonadotropin concentrations in agonadal children and adults. J Clin Endocrinol Metab. 1972;35:561‐564. 5052975 10.1210/jcem-35-4-561

[44] Andersson AM , Toppari J , Haavisto AM , et al. Longitudinal reproductive hormone profiles in infants: peak of inhibin B levels in infant boys exceeds levels in adult men. J Clin Endocrinol Metab. 1998;83:675‐681. 9467591 10.1210/jcem.83.2.4603

[45] Winter JS , Faiman C , Hobson WC , Prasad AV , Reyes FI . Pituitary‐gonadal relations in infancy. I. Patterns of serum gonadotropin concentrations from birth to four years of age in man and chimpanzee. J Clin Endocrinol Metab. 1975;40:545‐551. 1127071 10.1210/jcem-40-4-545

[46] Conte FA , Grumbach MM , Kaplan SL . A diphasic pattern of gonadotropin secretion in patients with the syndrome of gonadal dysgenesis. J Clin Endocrinol Metab. 1975;40:670‐674. 1127077 10.1210/jcem-40-4-670

[47] Ross JL , Loriaux DL , Cutler GB Jr . Developmental changes in neuroendocrine regulation of gonadotropin secretion in gonadal dysgenesis. J Clin Endocrinol Metab. 1983;57:288‐893. 6408110 10.1210/jcem-57-2-288

[48] Kuiri‐Hanninen T , Sankilampi U , Dunkel U . Activation of hypohtalmaic‐pituitary‐gonadal axis in infancy. Horm Res Paediatr. 2014;82:73‐80. 25012863 10.1159/000362414

[49] Lanciotti L , Cofini M , Leonardi A , Penta L , Esposito S . Up‐to‐date review about Minipuberty and overview on hypothalamic‐pituitary‐gonadal Axis activation in fetal and neonatal life. Front Endocrinol (Lausanne). 2018;9:410. 30093882 10.3389/fendo.2018.00410 PMC6070773

[50] Hines M , Spencer D , Kung KT , Browne WV , Constantinescu M , Noorderhaven RM . The early postnatal period, mini‐puberty, provides a window on the role of testosterone in human neurobehavioural development. Curr Opin Neurobiol. 2016;38:69‐73. 26972372 10.1016/j.conb.2016.02.008

[51] Watanabe G , Terasawa E . In vivo release of luteinizing hormone releasing hormone increases with puberty in the female rhesus monkey. Endocrinology. 1989;125:92‐99. 2661213 10.1210/endo-125-1-92

[52] Chongthammakun S , Claypool LE , Terasawa E . Ovariectomy increases in vivo luteinizing hormone‐releasing hormone release in pubertal, but not prepubertal, female rhesus monkeys. J Neuroendocrinol. 1993;5:41‐50. 8485542 10.1111/j.1365-2826.1993.tb00362.x

[53] Guerriero KA , Keen KL , Terasawa E . Developmental increase in kisspeptin‐54 release in vivo is independent of the pubertal increase in estradiol in female rhesus monkeys (*Macaca mulatta*). Endocrinology. 2012;153:1887‐1897. 22315444 10.1210/en.2011-1701 PMC3320265

[54] Garcia JP , Guerriero KA , Keen KL , Kenealy BP , Seminara SB , Terasawa E . Kisspeptin and neurokinin B signaling network underlies the pubertal increase in GnRH release in female rhesus monkeys. Endocrinology. 2017;158:3269‐3280. 28977601 10.1210/en.2017-00500 PMC5659687

[55] Chongthammakun S , Terasawa E . Negative feedback effects of estrogen on luteinizing hormone‐releasing hormone release occur in pubertal, but not prepubertal, ovariectomized female rhesus monkeys. Endocrinology. 1993;132:735‐743. 8425492 10.1210/endo.132.2.8425492

[56] Claypool LE , Watanabe G , Terasawa E . Effects of electrical stimulation of the medial basal hypothalamus on the in vivo release of luteinizing hormone‐releasing hormone in the prepubertal and peripubertal female monkey. Endocrinology. 1990;127:3014‐3022. 2249640 10.1210/endo-127-6-3014

[57] Claypool LE , Kasuya E , Saitoh Y , Marzban F , Terasawa E . N‐methyl D,L‐aspartate induces the release of luteinizing hormone‐releasing hormone in the prepubertal and pubertal female rhesus monkey as measured by in vivo push‐pull perfusion in the stalk‐median eminence. Endocrinology. 2000;141:219‐228. 10614642 10.1210/endo.141.1.7231

[58] Plant TM , Gay VL , Marshall GR , Arslan M . Puberty in monkeys is triggered by chemical stimulation of the hypothalamus. Proc Natl Acad Sci U S A. 1989;86:2506‐2510. 2648405 10.1073/pnas.86.7.2506 PMC286942

[59] Wennink JM , Delemarre‐van de Waal HA , Schoemaker R , Schoemaker H , Schoemaker J . Luteinizing hormone and follicle stimulating hormone secretion patterns in girls throughout puberty measured using highly sensitive immunoradiometric assays. Clin Endocrinol (Oxf). 1990;33:333‐344. 2123756 10.1111/j.1365-2265.1990.tb00498.x

[60] Wu FCW , Butler GE , Kelnar CJH , Stirling HF , Huhtaniemi I . Patterns of pulsatile luteinizing and follicle stimulating hormone secretion in prepubertal (midchildhood) boys and girls and patients with idiopathic hypogonadotrophic hypogonadism (Kallmann's syndrome): a study using an ultrasensitive time‐resolved immunofluorometric assay. J Clin Endocrinol Metab. 1991;72:1229‐1237. 1902843 10.1210/jcem-72-6-1229

[61] Rosenfield RL . The ovary and female sexual maturation. In: Sperling MA , ed. Pediatric Endocrinology. Philadelphia, PA: W.B. Saunders; 1996:329‐385.

[62] Terasawa E , Nass TE , Yeoman RR , Loose MD , Schultz NJ . Hypothalamic control of puberty in the female rhesus macaque. In: Norman RL , ed. Neuroendocrine Aspects of Reproduction. New York, NY: Academic Press; 1983:149‐182.

[63] Boyar RM , Rosenfeld RS , Kapen S , et al. Human puberty. Simultaneous augmented secretion of luteinizing hormone and testosterone during sleep. J Clin Invest. 1974;54:609‐618. 4852310 10.1172/JCI107798 PMC301594

[64] Wildt L , Marshall G , Knobil E . Experimental induction of puberty in the infantile female rhesus monkey. Science. 1980;207:1373‐1375. 6986658 10.1126/science.6986658

[65] Seminara SB , Messager S , Chatzidaki EE , et al. The GPR54 gene as a regulator of puberty. N Engl J Med. 2003;349:1614‐1627. 14573733 10.1056/NEJMoa035322

[66] de Roux N , Genin E , Carel J‐C , Matsuda F , Chaussain J‐L , Milgrom E . Hypogonadotropic hypogonadism due to loss of function of the KiSS1‐derived peptide receptor GPR54. Proc Natl Acad Sci U S A. 2003;100:10972‐10976. 12944565 10.1073/pnas.1834399100 PMC196911

[67] Topaloglu AK , Reimann F , Guclu M , et al. TAC3 and TACR3 mutations in familial hypogonadotropic hypogonadism reveal a key role for neurokinin B in the central control of reproduction. Nat Genet. 2009;41:354‐358. 19079066 10.1038/ng.306 PMC4312696

[68] Oakley AE , Clifton DK , Steiner RA . Kisspeptin signaling in the brain. Endocr Rev. 2009;30:713‐743. 19770291 10.1210/er.2009-0005 PMC2761114

[69] Clarke IJ , Caraty A . Kisspeptin and seasonality of reproduction. Adv Exp Med Biol. 2013;784:411‐430. 23550017 10.1007/978-1-4614-6199-9_19

[70] Herbison AE . Control of puberty onset and fertility by gonadotropin‐releasing hormone neurons. Nat Rev Endocrinol. 2016;12:452‐466. 27199290 10.1038/nrendo.2016.70

[71] Uenoyama Y , Inoue N , Maeda KI , Tsukamura H . The roles of kisspeptin in the mechanism underlying reproductive functions in mammals. J Reprod Dev. 2018;64:469‐476. 30298825 10.1262/jrd.2018-110 PMC6305848

[72] Herde MK , Iremonger KJ , Constantin S , Herbison AE . GnRH neurons elaborate a long‐range projection with shared axonal and dendritic functions. J Neurosci. 2013;33:12689‐12697. 23904605 10.1523/JNEUROSCI.0579-13.2013 PMC6618539

[73] Cheng G , Coolen LM , Padmanabhan V , Goodman RL , Lehman MN . The kisspeptin/neurokinin B/dynorphin (KNDy) cell population of the arcuate nucleus: sex differences and effects of prenatal testosterone in sheep. Endocrinology. 2010;151:301‐311. 19880810 10.1210/en.2009-0541 PMC2803147

[74] Goodman RL , Lehman MN , Smith JT , et al. Kisspeptin neurons in the arcuate nucleus of the ewe express both dynorphin A and neurokinin B. Endocrinology. 2007;148:5752‐5760. 17823266 10.1210/en.2007-0961

[75] Lehman MN , Coolen LM , Goodman RL . Minireview: kisspeptin/neurokinin B/dynorphin (KNDy) cells of the arcuate nucleus: a central node in the control of gonadotropin‐releasing hormone secretion. Endocrinology. 2010;151:3479‐3489. 20501670 10.1210/en.2010-0022 PMC2940527

[76] Goodman RL , Hileman SM , Nestor CC , et al. Kisspeptin, neurokinin B, and dynorphin act in the arcuate nucleus to control activity of the GnRH pulse generator in ewes. Endocrinology. 2013;154:4259‐4269. 23959940 10.1210/en.2013-1331 PMC3800763

[77] Lehman MN , He W , Coolen LM , Levine JE , Goodman RL . Does the KNDy Model for the control of gonadotropin‐releasing hormone pulses apply to monkeys and humans? Semin Reprod Med. 2019;37:71‐83. 31847027 10.1055/s-0039-3400254 PMC9097242

[78] Hrabovszky E , Sipos MT , Molnár CS , et al. Low degree of overlap between kisspeptin, neurokinin B, and dynorphin immunoreactivities in the infundibular nucleus of young male human subjects challenges the KNDy neuron concept. Endocrinology. 2012;153:4978‐4989. 22903610 10.1210/en.2012-1545 PMC3512020

[79] Skrapits K , Borsay BÁ , Herczeg L , Ciofi P , Liposits Z , Hrabovszky E . Neuropeptide co‐expression in hypothalamic kisspeptin neurons of laboratory animals and the human. Front Neurosci. 2015;9:29. 25713511 10.3389/fnins.2015.00029 PMC4322635

[80] Mitsushima D , Hei DL , Terasawa E . gamma‐Aminobutyric acid is an inhibitory neurotransmitter restricting the release of luteinizing hormone‐releasing hormone before the onset of puberty. Proc Natl Acad Sci U S A. 1994;91:395‐399. 8278400 10.1073/pnas.91.1.395 PMC42954

[81] Mitsushima D , Marzban F , Luchansky LL , et al. Role of glutamic acid decarboxylase in the prepubertal inhibition of the luteinizing hormone releasing hormone release in female rhesus monkeys. J Neurosci. 1996;16:2563‐7253. 8786432 10.1523/JNEUROSCI.16-08-02563.1996 PMC6578748

[82] Kasuya E , Nyberg CL , Mogi K , Terasawa E . A role of gamma‐amino butyric acid (GABA) and glutamate in control of puberty in female rhesus monkeys: effect of an antisense oligodeoxynucleotide for GAD67 messenger ribonucleic acid and MK801 on luteinizing hormone‐releasing hormone release. Endocrinology. 1999;140:705‐712. 9927297 10.1210/endo.140.2.6574

[83] Keen KL , Burich AJ , Mitsushima D , Kasuya E , Terasawa E . Effects of pulsatile infusion of the GABA(A) receptor blocker bicuculline on the onset of puberty in female rhesus monkeys. Endocrinology. 1999;140:5257‐5266. 10537156 10.1210/endo.140.11.7139

[84] Kurian JR , Keen KL , Guerriero KA , Terasawa E . Tonic control of kisspeptin release in prepubertal monkeys: implications to the mechanism of puberty onset. Endocrinology. 2012;153:3331‐3336. 22585828 10.1210/en.2012-1221 PMC3380308

[85] Garcia JP , Keen KL , Anderson RM , Hirshfeld BC , Seminara SB , Terasawa E . Prepubertal tonic gamma‐amino butyric acid (GABA) inhibition is upstream of neurokinin B (NKB), kisspeptin, and gonadotropin releasing hormone (GnRH) neuronal network in male rhesus monkeys. Abstracts of the 101st Annual Meeting of the Endocrine Society, held on March 23–26, 2019, at New Orleans, LA (No. P1‐411).

[86] Terasawa E , Luchansky LL , Kasuya E , Nyberg CL . An increase in glutamate release follows a decrease in gamma aminobutyric acid and the pubertal increase in luteinizing hormone releasing hormone release in the female rhesus monkeys. J Neuroendocrinol. 1999;11:275‐282. 10223281 10.1046/j.1365-2826.1999.00325.x

[87] Herbison AE , Moenter SM . Depolarising and hyperpolarising actions of GABA(A) receptor activation on gonadotrophin‐releasing hormone neurones: towards an emerging consensus. J Neuroendocrinol. 2011;23:557‐569. 21518033 10.1111/j.1365-2826.2011.02145.x PMC3518440

[88] Berg T , Silveira MA , Moenter SM . Prepubertal development of GABAergic transmission to gonadotropin‐releasing hormone (GnRH) neurons and postsynaptic response are altered by prenatal androgenization. J Neurosci. 2018;38:2283‐2293. 29374136 10.1523/JNEUROSCI.2304-17.2018 PMC5830516

[89] Abreu AP , Dauber A , Macedo DB , et al. Central precocious puberty caused by mutations in the imprinted gene MKRN3. N Engl J Med. 2013;368:2467‐2475. 23738509 10.1056/NEJMoa1302160 PMC3808195

[90] Perry JR , Stolk L , Franceschini N , et al. Meta‐analysis of genome‐wide association data identifies two loci influencing age at menarche. Nat Genet. 2009;41:648‐650. 19448620 10.1038/ng.386 PMC2942986

[91] Perry JR , Elks CE , Sulem P , et al. Parent‐of‐origin‐specific allelic associations among 106 genomic loci for age at menarche. Nature. 2014;514:92‐96. 25231870 10.1038/nature13545 PMC4185210

[92] Lomniczi A , Wright H , Castellano JM , et al. Epigenetic regulation of puberty via Zinc finger protein‐mediated transcriptional repression. Nat Commun. 2015;6:10195. 26671628 10.1038/ncomms10195 PMC4703871

[93] Abreu AP , Toro CA , Song YB , et al. MKRN3 inhibits the reproductive axis through actions in kisspeptin‐expressing neurons. J Clin Invest. 2020;130:4486‐4500. 32407292 10.1172/JCI136564 PMC7410046

[94] Mayer C , Acosta‐Martinez M , Dubois SL , et al. Timing and completion of puberty in female mice depend on estrogen receptor alpha‐signaling in kisspeptin neurons. Proc Natl Acad Sci U S A. 2010;107:22693‐22698. 21149719 10.1073/pnas.1012406108 PMC3012491

[95] Terasawa E , Levine JE . Neuroendocrine regulation of puberty. In: Pfaff DW , Joels M , eds. Molecular Mechanisms of Hormone Actions on Behavior, Volume 5: Development of Hormone‐Behavior Relationships. 3rd ed. New York, NY: Academic Press; 2016:309‐356.

[96] Norman RL , Spies HG . Cyclic ovarian function in a male macaque: additional evidence for a lack of sexual differentiation in the physiological mechanisms that regulate the cyclic release of gonadotropins in primates. Endocrinology. 1986;118:2608‐2610. 3084224 10.1210/endo-118-6-2608

[97] Watanabe Y , Uenoyama Y , Suzuki J , et al. Oestrogen‐induced activation of preoptic kisspeptin neurones may be involved in the luteinizing hormone surge in male and female Japanese monkeys. J Neuroendocrinol. 2014;26:909‐917. 25283748 10.1111/jne.12227

[98] Bano R , Shamas S , Khan SUH , Shahab M . Inverse age‐related changes between hypothalamic NPY and KISS1 gene expression during pubertal initiation in male rhesus monkey. Reprod Biol. 2022;22:100599. 35033902 10.1016/j.repbio.2021.100599

[99] El Majdoubi M , Sahu A , Ramaswamy S , Plant TM . Neuropeptide Y: a hypothalamic brake restraining the onset of puberty in primates. Proc Natl Acad Sci U S A. 2000;97:6179‐6184. 10811877 10.1073/pnas.090099697 PMC18578

[100] Lanfray D , Richard D . Emerging signaling pathway in arcuate feeding‐related neurons: role of the Acbd7. Front Neurosci. 2017;11:328. 28690493 10.3389/fnins.2017.00328 PMC5481368

[101] Keen KL , Wegner FH , Bloom SR , Ghatei MA , Terasawa E . An increase in kisspeptin‐54 release occurs with the pubertal increase in luteinizing hormone‐releasing hormone‐1 release in the stalk‐median eminence of female rhesus monkeys in vivo. Endocrinology. 2008;149:4151‐4157. 18450954 10.1210/en.2008-0231 PMC2488227

[102] Terasawa E , Garcia JP . Neuroendocrine mechanisms of puberty in non‐human primates. Curr Opin Endocr Metab Res. 2020;14:145‐151. 33043166 10.1016/j.coemr.2020.07.008 PMC7543877

[103] Garcia JP , Keen KL , Kenealy BP , Seminara SB , Terasawa E . Role of kisspeptin and neurokinin B signaling in male rhesus monkey puberty. Endocrinology. 2018;159:3048‐3060. 29982393 10.1210/en.2018-00443 PMC6456982

[104] Guerriero KA , Keen KL , Millar RP , Terasawa E . Developmental changes in GnRH release in response to kisspeptin agonist and antagonist in female rhesus monkeys (*Macaca mulatta*): implication for the mechanism of puberty. Endocrinology. 2012;153:825‐836. 22166978 10.1210/en.2011-1565 PMC3275383

[105] Messager S , Chatzidaki EE , Ma D , et al. Kisspeptin directly stimulates gonadotropin‐releasing hormone release via G protein‐coupled receptor 54. Proc Natl Acad Sci U S A. 2005;102:1761‐1766. 15665093 10.1073/pnas.0409330102 PMC545088

[106] Dumalska I , Wu M , Morozova E , Liu R , van den Pol A , Alreja M . Excitatory effects of the puberty‐initiating peptide kisspeptin and group I metabotropic glutamate receptor agonists differentiate two distinct subpopulations of gonadotropin‐releasing hormone neurons. J Neurosci. 2008;28:8003‐8013. 18685025 10.1523/JNEUROSCI.1225-08.2008 PMC2597556

[107] Krajewski SJ , Anderson MJ , Iles‐Shih L , Chen KJ , Urbanski HF , Rance NE . Morphologic evidence that neurokinin B modulates gonadotropin‐releasing hormone secretion via neurokinin 3 receptors in the rat median eminence. J Comp Neurol. 2005;489:372‐386. 16025449 10.1002/cne.20626

[108] Gaskins GT , Glanowska KM , Moenter SM . Activation of neurokinin 3 receptors stimulates GnRH release in a location‐dependent but Kisspeptin‐independent manner in adult mice. Endocrinology. 2013;154:3984‐3989. 23928373 10.1210/en.2013-1479 PMC3800761

[109] Garcia JP , Keen KL , Seminara SB , Terasawa E . Role of kisspeptin and NKB in puberty in nonhuman primates: sex differences. Semin Reprod Med. 2019;37:47‐55. 31847024 10.1055/s-0039-3400253 PMC7055930

[110] Ramaswamy S , Seminara SB , Plant TM . Evidence from the agonadal juvenile male rhesus monkey (*Macaca mulatta*) for the view that the action of neurokinin B to trigger gonadotropin‐releasing hormone release is upstream from the kisspeptin receptor. Neuroendocrinology. 2011;94:237‐245. 21832818 10.1159/000329045 PMC3238032

[111] Terasawa E , Fernandez DL . Neurobiological mechanisms of the onset of puberty in primates. Endocr Rev. 2001;22:111‐151. 11159818 10.1210/edrv.22.1.0418

[112] Terasawa E . Chongthammakun S Developmental Changes in In Vivo Release of β‐Endorphin (β‐END) from the Stalk‐Median Eminence (S‐ME) in Female Rhesus Monkeys. Abstracts of the 21st annual meeting of the neuroscience society, No. 361.1, New Orleans, LA. 1991

[113] Lippincott MF , León S , Chan YM , et al. Hypothalamic reproductive endocrine pulse generator activity independent of neurokinin B and dynorphin signaling. J Clin Endocrinol Metab. 2019;104:4304‐4318. 31132118 10.1210/jc.2019-00146 PMC6736049

[114] Keen KL , Petersen AJ , Figueroa AG , et al. Physiological characterization and transcriptomic properties of GnRH neurons derived from human stem cells. Endocrinology. 2021;162(9):bqab120. 34125902 10.1210/endocr/bqab120 PMC8294693

[115] Kenealy BP , Keen KL , Kapoor A , Terasawa E . Neuroestradiol in the stalk median eminence of female rhesus macaques decreases in association with puberty onset. Endocrinology. 2016;157:70‐76. 26496022 10.1210/en.2015-1770 PMC4701893

[116] Terasawa E . International neuroendocrine federation masterclass series: the GnRH neuron and its control, first edition. In: Herbison AE , Plant TM , eds. Postnatal Development of GnRH Neuronal Function. Hoboken, NJ: John Wiley & Sons; 2018:61‐91.

